# Jasmonic Acid-Dependent MYC Transcription Factors Bind to a Tandem G-Box Motif in the *YUCCA8* and *YUCCA9* Promoters to Regulate Biotic Stress Responses

**DOI:** 10.3390/ijms22189768

**Published:** 2021-09-09

**Authors:** Marta-Marina Pérez-Alonso, Beatriz Sánchez-Parra, Paloma Ortiz-García, Maria Estrella Santamaría, Isabel Díaz, Stephan Pollmann

**Affiliations:** 1 Centro de Biotecnología y Genómica de Plantas, Instituto Nacional de Investigación y Tecnología Agraria y Alimentación (INIA), Universidad Politécnica de Madrid (UPM), 28223 Pozuelo de Alarcón, Spain; beatriz.sanchez-parra@uni-graz.at (B.S.-P.); p.ortiz@upm.es (P.O.-G.); me.santamaria@upm.es (M.E.S.); i.diaz@upm.es (I.D.); 2 Department of Forest Genetics and Plant Physiology, Umeå Plant Sciences Centre (UPSC), Swedish University of Agricultural Science, 90183 Umeå, Sweden; 3 Institut für Biologie, Bereich Pflanzenwissenschaften, Karl-Franzens Universität Graz, 8010 Graz, Austria; 4 Departamento de Biotecnología-Biología Vegetal, Escuela Técnica Superior de Ingeniería Agronómica, Alimentaria y de Biosistemas, Universidad Politécnica de Madrid (UPM), 28040 Madrid, Spain

**Keywords:** *Arabidopsis thaliana*, indole-3-acetic acid, jasmonic acid, plant hormone crosstalk, transcriptional regulation, wound response, biotic stress, growth-defense trade-off

## Abstract

The indole-3-pyruvic acid pathway is the main route for auxin biosynthesis in higher plants. Tryptophan aminotransferases (TAA1/TAR) and members of the YUCCA family of flavin-containing monooxygenases catalyze the conversion of l-tryptophan via indole-3-pyruvic acid to indole-3-acetic acid (IAA). It has been described that jasmonic acid (JA) locally produced in response to mechanical wounding triggers the de novo formation of IAA through the induction of two *YUCCA* genes, *YUC8* and *YUC9*. Here, we report the direct involvement of a small number of basic helix-loop-helix transcription factors of the MYC family in this process. We show that the JA-mediated regulation of the expression of the *YUC8* and *YUC9* genes depends on the abundance of MYC2, MYC3, and MYC4. In support of this observation, seedlings of *myc* knockout mutants displayed a strongly reduced response to JA-mediated IAA formation. Furthermore, transactivation assays provided experimental evidence for the binding of MYC transcription factors to a particular tandem G-box motif abundant in the promoter regions of *YUC8* and *YUC9*, but not in the promoters of the other *YUCCA* isogenes. Moreover, we demonstrate that plants that constitutively overexpress *YUC8* and *YUC9* show less damage after spider mite infestation, thereby underlining the role of auxin in plant responses to biotic stress signals.

## 1. Introduction

Since its discovery in the 1930s [1,2,3], many studies have firmly demonstrated that auxins govern virtually every aspect of plant life related to growth and development, e.g., cell elongation, tropisms, apical dominance, initiation of lateral and adventitious root growth, senescence, and flowering [4,5,6]. However, despite the great importance of auxin for plant development, the role of auxin in plant defense is still not fully understood. Several pieces of evidence indicate that indole-3-acetic acid (IAA), the major auxin in plants, exerts a negative role in resistance to plant stress, and that investing in plant growth imposes a penalty on plant defense, and vice versa [7,8,9,10]—a phenomenon referred to as the growth-defense trade-off [11]. Consistent with these affirmations, activation of IAA-mediated stem elongation in response to light has been described to make *Arabidopsis thaliana* and *Chenopodium album* plants more susceptible to different pathogens, such as the bacteria *Pseudomonas syringae* or larvae of the beet armyworm *Spodoptera exigua* [12,13]. Similarly, Mutka and co-workers [14] showed that an increase in endogenous IAA levels in Arabidopsis decreases plant tolerance to *P. syringae*. On the contrary, another series of studies suggests that auxin can positively influence plant tolerance. Here, the auxin signaling component AUXIN RESPONSE FACTOR 3 (ARF3) has been reported to control the formation of leaf trichomes, considered a direct defense mechanism against predators [15,16]. In addition, analyses carried out using the Arabidopsis auxin reporter line DR5::GUS showed that mechanical wounding stimulates IAA signaling in neighboring unwounded plants [17], suggesting an indirect defense mechanism that allows plants to prepare for a possible imminent attack. With the aim of adding to this knowledge, we intend not only to shed light on the implication of auxin in plant biotic stress responses, but also to provide new molecular targets that contribute to the plant growth-defense trade-off phenomenon.

In the context of plant stress responses, jasmonates play a prominent role. They are lipid-derived hormones comprising jasmonic acid (JA) and several derivatives of JA [18]. These molecules play an essential role in counteracting abiotic and biotic stress responses, such as pathogen and herbivore attacks [19,20,21]. Upon recognition of stress, the production of the bioactive JA, jasmonoyl-l-isoleucine (JA-Ile) is stimulated and perceived by the CORONATINE INSENSITIVE 1 (COI1) protein. This enables the Skp-Cullin 1-F-box (SCF) E3 ligase complex to interact with and ubiquitinate specific repressor proteins known as JASMONATE ZIM DOMAIN proteins (JAZ). Degradation of JAZs by the 26S proteasome releases the MYC family of basic helix-loop-helix (bHLH) transcription factors from repression, which subsequently triggers the expression of different subsets of genes responsive to JA [11,22,23].

In recent years, a wide range of links between the JA and auxin signaling pathways have been reported. For example, JA has been shown to negatively affect primary root growth in *A. thaliana* through transcriptional repression of *PLETHORA* genes, namely *PLT1* and *PLT2*, which are known as essential transcription factors that control the specification and maintenance of the auxin-regulated root meristem [24]. More recent work has described a crosstalk model in which wound-inducible amidohydrolases contribute to the cellular regulation of JA and auxin levels to coordinate stress responses by controlling JA- and IAA-amino acid conjugate contents [16]. On the contrary, a series of publications emphasized that JA exerts a direct stimulating effect on various phases of auxin biosynthesis. Dombrecht et al. [25] reported the JA-associated transcription factor MYC2 to control the formation of auxin biosynthesis precursors as well as auxin-related defense compounds, including indole glucosinolates. Furthermore, JA has been demonstrated to promote auxin de novo-biosynthesis through the transcriptional activation of two anthranilate synthase genes, *ASA1* and *ASB1*, resulting in elevated levels of l-tryptophan and, therefore, increased precursor availability for auxin biosynthesis [26]. Hentrich et al. [27] have reported a more direct impact on auxin biosynthesis. The authors provided evidence for the JA-dependent transcriptional activation of two Arabidopsis *YUCCA* genes, *YUC8* and *YUC9*. Intriguingly, YUCCA enzymes are considered key players in general IAA biosynthesis, along with tryptophan aminotransferases [28,29,30,31]. Importantly, the gene expression studies presented by Hentrich et al. [27] showed that the transcriptional response of *YUC8* and *YUC9* to the treatment with different oxylipins, including methyl jasmonate (MeJA) and its precursor 12-oxo-phytodienoic acid (OPDA), is almost entirely impaired in the *coi1* mutant background. Based on this experiment, it was speculated that the transcriptional regulation of *YUC8* and *YUC9* depends on the COI-JAZ-MYC signaling module.

In this work, we show that the Arabidopsis transcription factor MYC2, and its closest homologues MYC3 and MYC4 [32], play a direct role in the regulation of auxin biosynthesis through the control of the expression of the *YUC8* and *YUC9* genes. We demonstrate that different *myc* knockout mutants display a significant reduction not only in JA-mediated IAA production, but also in the accumulation of *YUC8* and *YUC9* transcripts. Moreover, our transient transactivation analyses in *Nicotiana benthamiana* and Arabidopsis protoplasts clearly demonstrate that MYC2, MYC3, and MYC4 bind to a specific tandem G-box motif abundant in the promoter regions of *YUC8* and *YUC9*, which is absent in the promoters of the other *YUCCA* genes. Finally, *YUC8* and *YUC9* promoter-reporter lines and mutants have been subjected to biotic stress conditions by applying the two-spotted spider mite *Tetranychus urticae* to their leaves, which provided compelling evidence that the overexpression of the *YUC9* gene rendered the mutant plants more resistant to the herbivore predators. Taken together, our results provide evidence for a signal transduction mechanism that employs the COI-JAZ-MYC module to fine-tune the expression of auxin biosynthesis-related genes in response to wounding and resistance to phytophagous mites.

## 2. Results

### 2.1. MYC2, MYC3, and MYC4 Trigger Auxin Formation after JA Treatment

**Figure 1:**
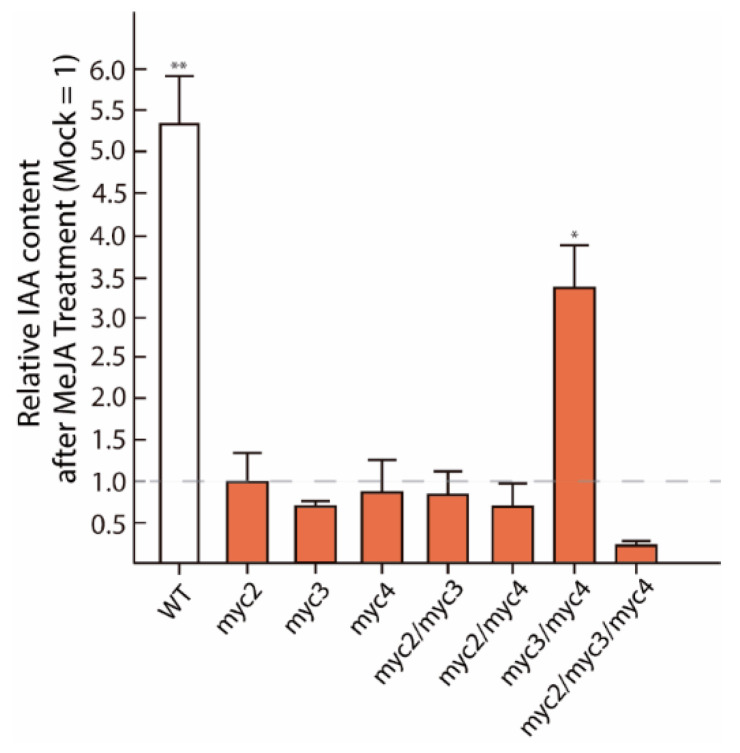
JA-triggered IAA biosynthesis is curtailed in *myc* mutants. IAA contents were assessed by GC-MS/MS in 10 days-old seedlings treated with either 50 µM MeJA or a mock solution (0.5% MeOH (*v*/*v*)) for 4 h. Stable isotope labelled [^2^H_2_]-IAA was used as internal standard for the absolute quantification of IAA in the samples. To determine the relative IAA-production after MeJA-treatment, the IAA contents in the mock treated samples were arbitrarily set to a value of one and the IAA content in MeJA-treated samples was expressed relative to this value. The bars show the mean ± SE (*n* = 3). Significant differences between means, comparing mock treated seedlings with the respective treated WT or *myc* loss-of-function mutant, are indicated with asterisks (* *p* < 0.05, ** *p* < 0.01; Student’s *t*-test).

First, we investigated whether MYC transcription factors are directly involved in de novo IAA synthesis. To this end, we quantified the endogenous IAA levels in *A. thaliana* wild-type seedlings (Col-0), as well as different *myc* mutants, four hours after the treatment with 50 µM MeJA by gas chromatography-tandem mass spectrometry (GC-MS/MS) (Figure 1). Confirming previous observations [27], the application of MeJA to WT seedlings translated into a more than 4.5-fold increased IAA formation relative to mock-treated control seedlings (0.5% MeOH (*v*/*v*)). On the contrary, there was no detectable stimulation of IAA production in *myc* single mutants, the *myc2/myc3* and *myc2/myc4* double mutants, and the *myc2/myc3/myc4* triple mutant upon MeJA-treatment. Interestingly, we observed a pronounced reduction in IAA accumulation in the *myc* triple mutant (0.5-fold) when compared with the respective mock-treated control. This observation prompts us to think that the three MYCs transcription factors collaborate in the control of JA-mediated IAA accumulation. Remarkably, although all single *myc* mutants displayed a general tendency of impaired MeJA-mediated IAA formation (no significant differences to the mock-treated controls), *myc3/myc4* seedlings exhibited a remaining significant increase in IAA contents in response to the MeJA application. Intriguingly, a recent study demonstrated the overexpression of the *GROWTH REGULATING FACTOR (GRF)-INTERACTION FACTOR 1* (GIF1) in the double *myc3/myc4* mutant [33] This transcriptional co-activator regulates leaf growth and development together with GRFs, a group of transcription factors that could contribute to the regulation of auxin biosynthesis [34,35]. However, with an increase of approximately 3.5-fold over the corresponding mock-treated *myc3/myc4* seedlings, the detected response was still weaker than the one observed for wild-type seedlings.

In summary, the presented results support the idea of an intimate crosstalk between JA signaling and IAA biosynthesis. At the same time, taking the strongest wounding response of MYC2 relative to the other two MYCs into account [32], the obtained data suggest the participation of all tested MYC proteins in the regulation of *YUC8/9* gene expression, and point toward a possibly leading role of MYC2 in this process.

### 2.2. MeJA-Dependent YUC8 and YUC9 Induction Is Abolished in Myc Loss-of-Function Mutants

**Figure 2:**
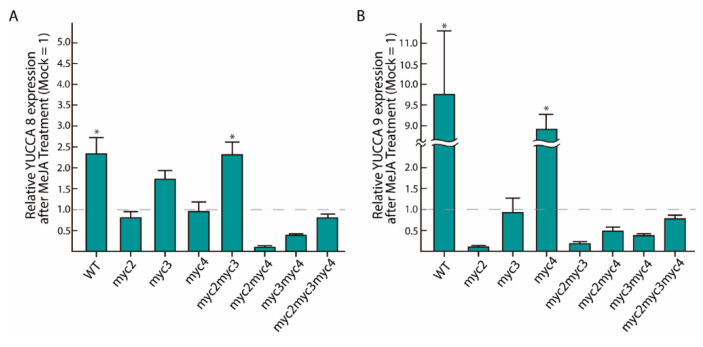
MYC2, MYC3, and MYC4 contribute to the control *YUC8* and *YUC9* expression in response to MeJA. Depicted is the qRT-PCR analysis of (**A**) *YUC8* and (**B**) *YUC9* expression after MeJA-treatment (4 h and 2 h for *YUC8* and *YUC9*, respectively). The transcript levels of *YUC8* and *YUC9* are given relative to the reference genes *UBI10* and *APT1* and normalized using the mock treated seedlings (0.5% MeOH (*v*/*v*)). The data shown are mean ± SE (*n* = 3). We established a two-fold change between the mock treated seedlings and the respective MeJA-treated seedlings as threshold to assume a differential regulation (* fold-change ≥ 2).

To confirm the role of MYC transcription factors in the transcriptional activation of *YUC8* and *YUC9*, we conducted quantitative reverse transcriptase PCR (qRT-PCR) analyses after seedlings were treated with exogenous MeJA. In accordance with previous results, we observed that *YUC8* and *YUC9* expression increased 1.3-fold and 8.7-fold in WT seedlings, respectively (Figure 2). In addition, we found that the gene expression of both *YUC* genes was unaffected by the exogenous application of MeJA in practically all single, double, and triple *myc* knockout mutants. Surprisingly, in both *myc3* and *myc2/myc3* the *YUC8* transcript accumulation was slightly activated after 4 h of MeJA treatment (0.67-fold induction and 1.4-fold induction, respectively) (Figure 2A). Notably, in the *myc4* background *YUC9* expression was significantly induced after 4 h of MeJA application (7.8-fold induction) (Figure 2B). Consistent with this observation, Zhang et al. [36] demonstrated a significantly higher JA accumulation in the *myc4* mutant after wounding, when compared to wt and the single *myc2* and *myc3* knockout lines. Taken together, these results underpin that MYC2 and MYC4 contribute to MeJA-induced *YUC8* transcription. While MYC2 and MYC3 are likely to contribute to the regulation of *YUC9* gene expression under this condition. It is noteworthy that we detected no complete loss of *YUC8* and *YUC9* expression in *myc2/myc3/myc4* (Figure 2A,B). This observation may be interpreted as an indication for a compensatory mechanism to attenuate the absence of JA response in plants or the involvement of other MYC paralogs, such as MYC5, which has recently been reported to contribute to plant defense [37]. However, the transcriptomics analysis of the *myc2/myc3/myc4* triple mutant provided no evidence for upregulation of *MYC5* gene expression [38].

### 2.3. The YUC8 and YUC9 Promoters Contain a Conserved MYC2, MYC3, and MYC4 Binding Motif

**Figure 3:**
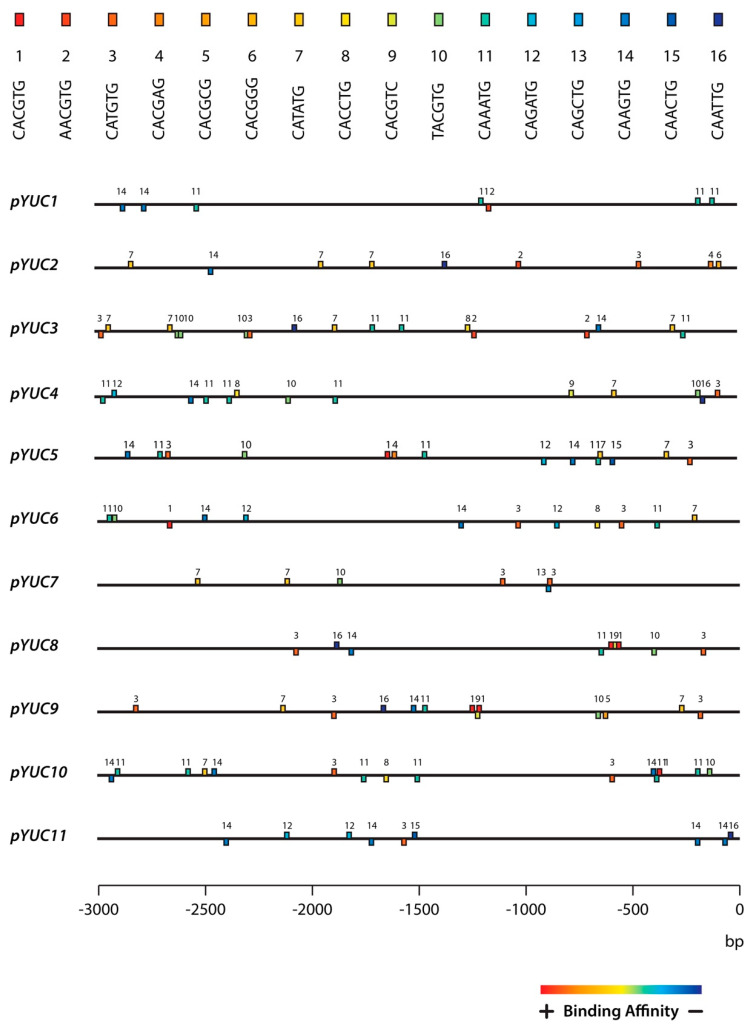
The promoters of *YUC8* and *YUC9* present a specific G-boxes binding motif configuration. Schematic representation of the distribution of G-box and putative G-box variants in the promoter of *Arabidopsis YUCCA* genes. The -3000 to -1 promoter region upstream to the transcriptional start codon (ATG) of the eleven *YUC* family members is shown. All reported G-boxes are color-coded (square) and associated with a specific sequence. Numbers indicate the corresponding G-box nucleotide sequence, with number 1 being the described canonical G-box. Colors indicate different experimental MYC2 binding affinities [32].

To further investigate the role of MYC2, MYC3, and MYC4 in the transcriptional regulation of *YUC8* and *YUC9*, the 3000 bp sequence upstream of the transcription start-codon was retrieved for the eleven *A. thaliana YUCCA* genes and used to screen for MYC binding motifs, i.e., for the canonical G-box (5′CACGTG-3′) and its fifteen described G-box variants [32]. As presented in Figure 3, we noticed that all *YUCCA* promoters contained a considerable number of JA-responsive elements. Most remarkable, however, was the observation that only the promotors of *YUC8* (*pYUC8*) and *YUC9* (*pYUC9*) contain a particular “tandem” DNA binding motif configuration. This “tandem” consisted in two canonical G-boxes (5’-CACGTG-3’), designated with number 1 in Figure 3, and one G-box variant 9 (5’-CACGTC-3’) at the nucleotide positions -535, -555 and -571 in case of *pYUC8*, and -1240, -1247 and -1272 in *pYUC9*. Furthermore, we found that the observed “1-9-1” configuration is accompanied by the G-box variant 3 (5’-CATGTG -3’) in positions -140 and -207 of *pYUC8* and *pYUC9*, respectively.

Notably, the G-box variant 3 was relatively close to a 5’-TATAAA-3’ sequence, in positions -153 (*pYUC8*) and -267 (*pYUC9*). This sequence has been identified as the consensus TATA-box, a well-known transcriptional enhancer [39]. For this reason, we hypothesized that the observed combination of *cis*-elements (G-boxes) may be crucial for the transcriptional regulation of *YUC8* and *YUC9*, differentiating them from the other *YUC* genes.

**Figure A1:**
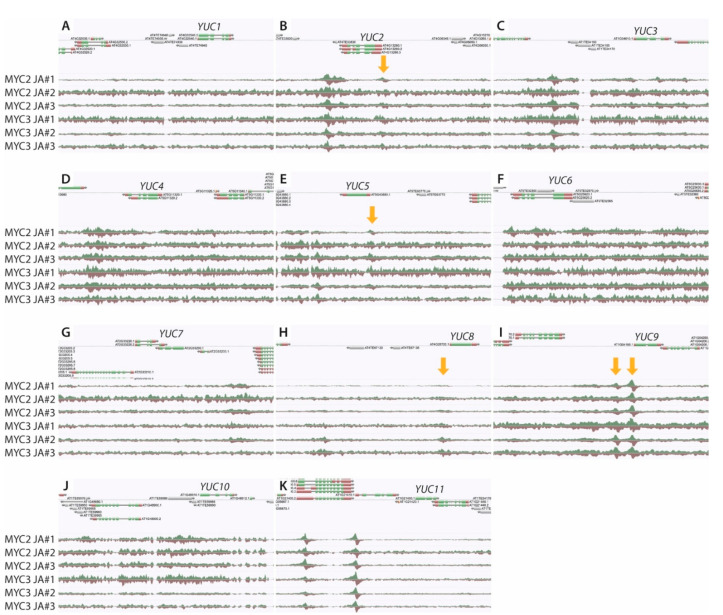
Genome browser screenshots showing the binding of MYC2 and MYC3 to the promoter regions of the eleven *YUCCA* genes. The figure shows the genomic regions around (**A**) *YUC1*, At4g32540; (**B**) *YUC2*, At4g13260; (**C**) *YUC3*, At1g04610; (**D**) *YUC4*, At5g11320; (**E**) *YUC5*, At5g43890; (**F**) *YUC6*, At5g25620; (**G**) *YUC7*, At2g33230; (**H**) *YUC8*, At4g28720; (**I**) *YUC9*, At1g04180; (**J**) *YUC10*, At1g48910; and (**K**) *YUC11*, At1g48910. The corresponding *YUCCA* genes are marked in each panel. Possible binding sites, represented by an enrichment of sequence reads obtained from either MYC2 or MYC3 ChIP-seq assays, in the *YUC* promoters are highlighted by arrows. The data have been extracted from Zander et al. [40]. Array results are available under: http://neomorph.salk.edu/MYC2, last accessed: 8 September 2021.

Intriguingly, our observations were recently partially confirmed by chromatin immunoprecipitation DNA-sequencing (ChIP-seq) assays using JA-treated Col-0 *MYC2::MYC2-YPet* and Col-0 *MYC3::MYC3-YPet* seedlings [40]. As can be taken from Figure A1, the AnnoJ genome browser screenshots visualize the binding of MYC2 and MYC3 particular to the promoter of *YUC9* and, to a lesser extent, to *YUC8*. In addition, there might be a less pronounced binding of MYC2 to the G-box #3 region of *YUC2* and *YUC5*, located close to the transcriptional start-codon. 

### 2.4. MYC2 Regulates YUC8 and YUC9 Expression through the Interaction with G-Box Elements

**Figure 4:**
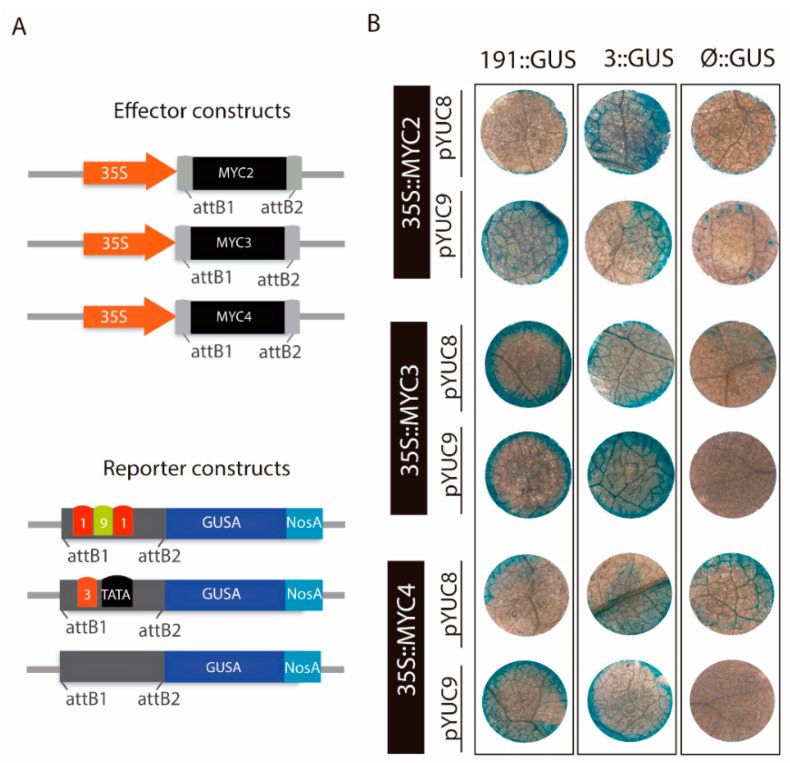
Transactivation of the *pYUC8::GUS* and *pYUC9::GUS* by MYC2, MYC3, and MYC4 in agroinfiltrated *N. benthamiana* leaf discs. (**A**) Schematic representation of the effector and reporter constructs used in the transient expression experiment. The effector constructs contain the CaMV *35S* promoter fused to the *MYC2*, *MYC3,* and *MYC4* ORFs. The reporter constructs contain different combinations of the G-box binding sites found in the *YUC8* and *YUC9* promoters, i.e., the tandem 1-9-1 (5’-CACGTG-CACGTC-CACGTG-3’)—the final *cis*-regulatory G-box #3 (5’-CATGTG-3’). Moreover, *Ø* refers to the promoter fragment lacking any of the mentioned DNA binding sites. All reporter constructs were fused to the *GUS* reporter gene, followed by the *NOS* terminator cassette. (**B**) Histochemical GUS staining of *N. benthamiana* leaf discs independently agroinfiltrated with the *35S::MYC2*, *35S::MYC3* and *35S::MYC4* constructs, and the reporter constructs -*191::GUS* and -*3::GUS* from the *pYUC8/9*. The -*Ø::GUS* constructs were used as a negative controls.

**Figure A2:**
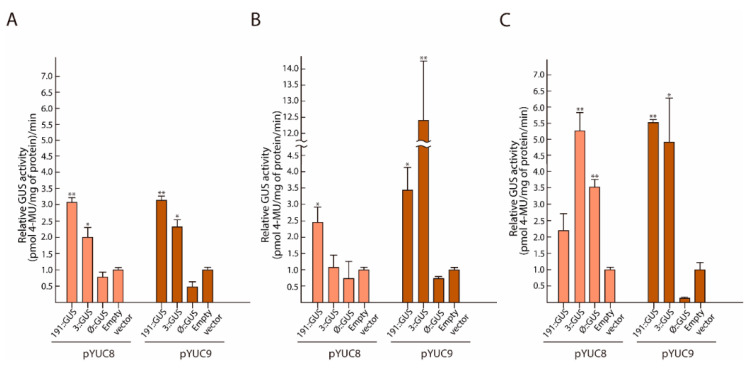
Relative GUS enzymatic activity in agroinfiltrated *N. benthamiana* leaves. (**A**) Fluororimetric GUS quantification of the -*191::GUS*, -*3::GUS* and -*Ø::GUS* reporter constructs co-transformed with the effector construct *35S::MYC2*, (**B**) *35S::MYC3*, and (**C**) *35S::MYC4*. In this case, the empty pMDC163 vector was generated by the deletion of the *ccdB* operon [92] and used as negative control. GUS activity was then normalized according to the empty vector, final units pmol 4-methylumelliferone (MU)/mg protein/min. Protein quantity was investigated using Bradford method [82]. Data are mean ± SE. Three different young leaves per plant were agroinfiltrated. GUS activation of each promoter construct was quantified in triplicates. Asterisks indicate significant differences between means (* *p* < 0.05, ** *p* < 0.01, Student’t *t*-test comparing negative control and independent promoter constructs).

Next, we studied whether the observed *cis*-regulatory elements are indeed involved in the transcriptional regulation of *YUC8* and *YUC9*. To this end, we performed a transient transactivation effector-reporter experiment in *N. benthamiana* leaves. To set up the effector plasmids, i.e., *35S::MYC2*, *35S::MYC3*, and *35S::MYC4*, the open reading frames from *MYC2*, *MYC3,* and *MYC4* were independently amplified and fused to the *Cauliflower mosaic virus* (CaMV) *35S* promoter (Figure 4A). To prove our hypothesis described above, we generated three reporter constructs for *pYUC8*, termed -*191::GUS*, -*3::GUS*, and -*Ø::GUS*, and three promoter constructs for *pYUC9*, referred to as -*191::GUS*, -*3::GUS*, and -*Ø::GUS* (Figure 4A). The different truncated promoter fragments for *YUC8* or *YUC9*, containing the tandem DNA motifs (191) or the final *cis*-acting element (#3), as well as a promoter segment without any of these regulatory sequences (Ø), were amplified by PCR and cloned into a vector carrying the *GUS* reporter gene. Subsequently, we investigated the transient *GUS* activation after *Agrobacterium*-mediated *N. benthaminana* leaf infiltration (Figure 4B). Remarkably, the experiment revealed that MYC2, MYC3, and MYC4 are capable of triggering *GUS* expression in presence of the -*191::GUS* and -*3::GUS* constructs of both *YUCCA* promoters studied, while the corresponding -*Ø::GUS* constructs served as negative controls. Nonetheless, we observed a faint patchy blue distribution in the -*Ø::GUS* leaf discs, indicating a very weak background activity of the constructs. In view of this result, we aimed at a quantitative assessment of *GUS* transactivation (Figure A2). In agreement with the results presented above, we observed that in presence of MYC2, the GUS activity of -*191::GUS* and -*3::GUS* samples increased 2-fold and 0.8-fold for the *pYUC8* constructs, and 2.3-fold and 1.3-fold for the *pYUC9* constructs, respectively when compared to the negative control (empty vector). Intriguingly, the quantitative analysis indicated that only the *pYUC8*-*191::GUS* construct was significantly activated when MYC3 was present. The lack of GUS activity in -*3::GUS* may indicate that MYC3 does not effectively bind to the 5’-CATGTG-3’ regulatory element #3. Nonetheless, the *pYUC9* results called this interpretation into question, since the fluorometric assay showed significant activation of the GUS activity in -*191::GUS* (2.5-fold) and -*3::GUS* (11.5-fold) relative to the negative control. Finally, we detected that only MYC4 activated the *pYUC8*-*3::GUS* construct, whereas, GUS activity levels were elevated in both *pYUC9*-*191::GUS* and *pYUC9*-*3::GUS,* 4.5-fold and 4-fold, respectively in comparison to the negative control (Figure A2). In contrast to MYC2 and MYC3, co-infiltration of *N. benthamiana* leaf discs with MYC4 resulted in a moderate GUS activity for the *pYUC8*-*Ø::GUS* construct. The analysis of the *YUC8* promoter revealed the presence of the 5′-CAAATG-3′ the G-box variant #11, suggesting that these DNA binding sites could be important in the transcriptional regulation of *YUC8* driven by MYC4. Overall, our analyses identified MYC2 as a positive regulator of the auxin biosynthesis-related genes *YUC8* and *YUC9*, most probably through its interaction with the promoter G-box “tandem” 1-9-1 and/or the G-box #3. With respect to the work of Sun et al. [26], the *YUC2* gene could also be a MYC2 target in Arabidopsis roots, probably through the G-box #3. In addition, our observations suggest that MYC3 and MYC4 may co-operate to control the expression of *YUC8* and *YUC9*.

**Figure 5:**
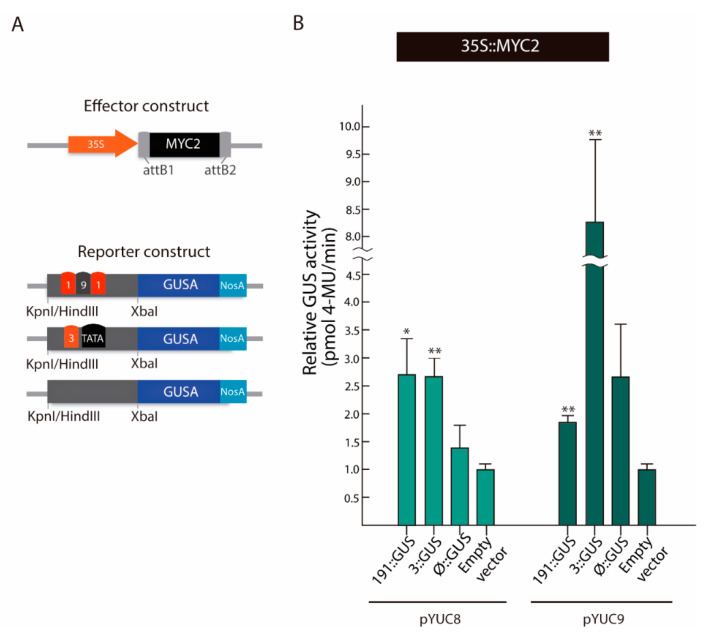
Transcriptional activity assay in *A. thaliana* mesophyll protoplasts. (**A**) Schematic representation of the effector constructs *35S::MYC2* and the -*191::GUS*, -*3::GUS* and -*Ø::GUS* reporter constructs used. (**B**) Fluorometric GUS activity quantification. The empty plasmid was employed as negative control. Here, GUS activity was relativized to the *NAN* reporter gene activity and normalized to the empty vector. Final GUS activation levels are expressed as pmol 4-methylumelliferone (MU)/min. Values are mean ± SE. To perform this experiment, three aliquots per protoplast suspension were inspected. Similar results were obtained in two independent experiments. Asterisks indicate Student’s *t*-test significant differences (* *p* < 0.05, ** *p* < 0.01).

To further validate the physical interaction between MYC2 and the *YUC8* and *YUC9* promoters by an alternative in planta method, we carried out a third experiment in *A. thaliana* mesophyll protoplasts (Figure 5). Here, constructs analogous to those used for the *N. benthamiana* transient expression assay were utilized. In addition, an empty pBT-10 plasmid was co-transfected with the *35S::MYC2* effector constructs as negative control (Figure 5A). Confirming our previous findings, the relative enzymatic GUS activities showed that, in comparison to the empty vector (named as negative control), MYC2 significantly activated the *GUS* reporter gene of the *pYUC8/9*-*191::GUS* and *pYUC8/9*-*3::GUS* constructs (Figure 5B). Notably, the observed GUS activity for the *pYUC9*-*Ø::GUS* construct exhibited an increment of approximately 1.5-fold, relative to the negative control. Like in the *N. benthamiana* transactivation assay; this could be due to the *cis*-regulatory elements found in the *Ø* promoter fragment (G-box variants 5 (5’-CACGCG-3’) and 10 (5’-TACGTG-3’)).

Most importantly, our results support the notion that the tested MYC protein in this assay, MYC2 directly binds to the G-box elements found in the *YUC8* and *YUC9* promoter, thereby controlling their gene expression.

### 2.5. YUC9 Plays a Role in Biotic Stress Responses

**Figure 6:**
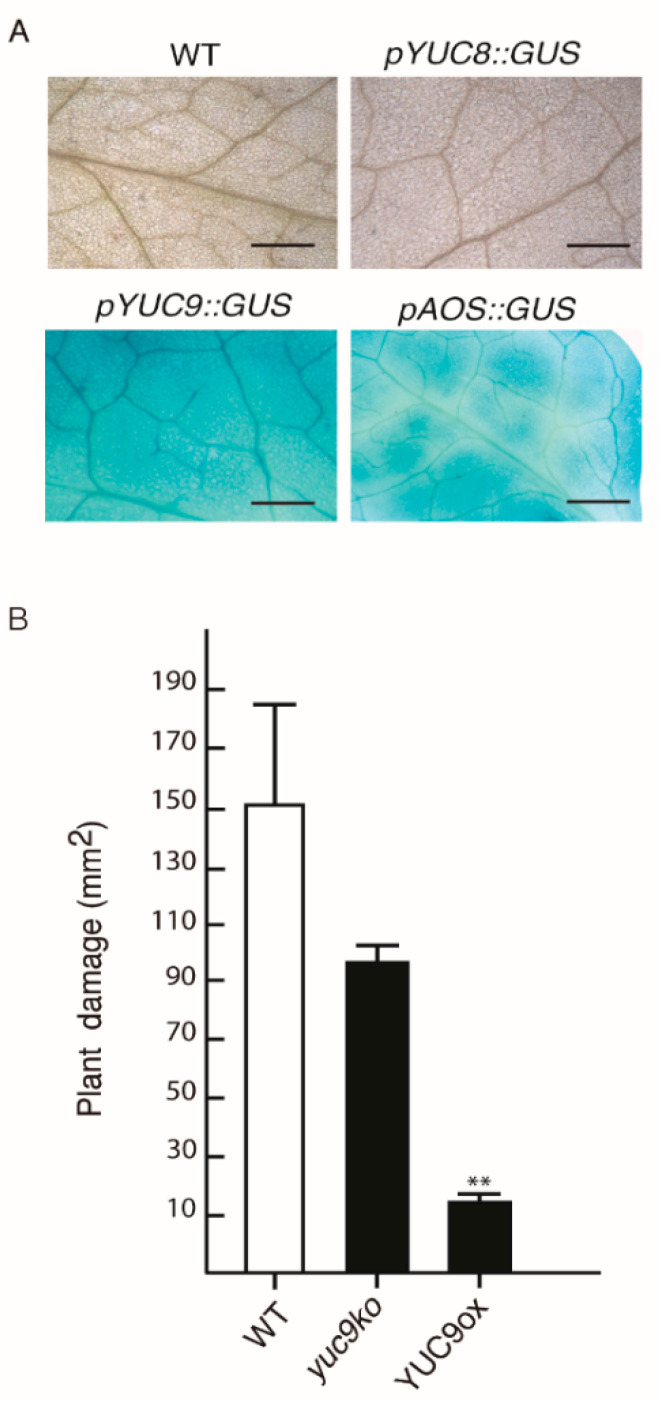
*YUC9* activation and plant damage assay after four days of spider mite (*Tetranychus urticae*) herbivory. (**A**) Histochemical GUS staining of leaves from wild-type *Arabidopsis*, *pYUC8::GUS*, *pYUC9::GUS* and *pAOS::GUS* plants, using 20 females from *T. urticae* per plant (*n* = 5). Scale bar = 50 μm. (**B**) Quantification of the total plant damage area (expressed in mm^2^) in WT, *yuc9ko* and YUC9ox mutant lines. Represented are means ± SE (*n* = 5). Student’s *t*-test: ** *p* < 0.01.

**Figure A3:**
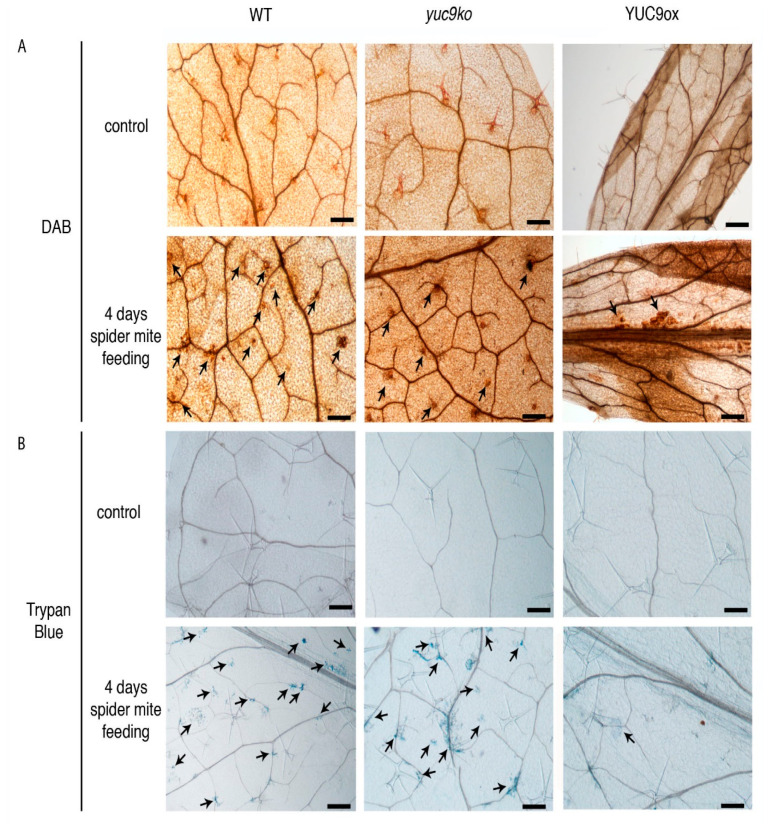
Histochemical analysis after spider mite herbivory. (**A**) DAB staining, (**B**) Trypan blue staining. Arrows indicate H_2_O_2_ accumulation and cell death, respectively. Images represent leaf details of control plants (top row) and plants exposed to 4 days spider mite feeding (bottom row). Scale bar = 1 mm. The arrows mark tissue damages caused by mite feeding.

The bHLH transcription factor MYC2 plays a key role in JA-mediated defense responses against herbivores and necrotrophic pathogens [23,41,42,43]. This prompted us to investigate the activation of *YUC8* and *YUC9* expression by a phytophagous pest. To this end, three to four weeks-old wild-type plants and the reporter lines *pYUC8::GUS, pYUC9::GUS,* and *pAOS::GUS* were exposed to the two-spotted spider mite, *Tetranychus urticae*. Subsequent GUS staining clearly revealed a strong reporter activity for the positive control, the *AOS* (At5g42650) promoter line, and the *YUC9* promoter driven construct (Figure 6A). On the contrary, the absence of visible GUS activity in *pYUC8::GUS* leaves subjected to *T. urticae* suggests that *YUC8* is possibly not involved in the defense against pests or that the response of *YUC8* is slower than the response of *YUC9*, which has previously been suggested for oxylipin treatments by Hentrich et al. [27]. In view of this result, we intended to shed light on the biological meaning of MYC2 driven auxin synthesis. To analyze if genetic alterations in *YUC9* expression have an influence on the susceptibility of the corresponding plants toward herbivorous predators, WT, YUC9 overexpressing plants (YUC9ox), and the *yuc9* mutant (*yuc9ko*) were tested. Twenty adult female spider mites were placed on single leaves of ten plants from each genotype and allowed to feed for four days. The leaf damage quantification highlighted a preference for adult mites to feed on WT rather than YUC9ox plants. This is displayed by an approximately 40% lower leaf damage area of YUC9ox compared to WT (Figure 6B). Interestingly, the *yuc9ko* mutants exhibited a decreased leaf damage area in comparison to WT, but the difference is statistically insignificant. Consistent with these observations, the 3,3′-diaminobenzidine (DAB) staining, which indicates the presence of H_2_O_2_, and the trypan blue exclusion test, which gives account on cell viability, determined a visibly higher accumulation of reactive oxygen species (ROS) and cell death in WT and *yuc9ko* plants in comparison to YUC9ox (Figure A3; Appendix A, Appendix methods A1 and A2).

## 3. Discussion

The existence of an intimate interplay between JA and IAA is highlighted by the fact that both phytohormones share a conserved signal transduction mechanism [44,45,46]. In line with this finding, Tiryaki and Staswick [47] demonstrated that depletion of the ubiquitination-related gene *AXR1* in Arabidopsis not only confers auxin resistance, but also generates MeJA insensitive mutants. This invited to think that AXR1 may contribute to the perception of both JA and IAA. In addition, evidence has been provided that a point mutation in one subunit forming the SCF-E3 ligase complex of Arabidopsis is enough to significantly reduced the transcript accumulation of JA-related genes and curtail the auxin response [48]. Downstream in the signaling cascade, both hormones cooperate spatiotemporally to regulate flower development and fertility through the action of ARF6 and ARF8 [49]. In rice coleoptiles, asymmetrical growth in response to gravitropism is simultaneously controlled by IAA-JA gradients [50]. On the other hand, crosstalk between JA signaling and IAA biosynthesis pathways has also been disclosed. Mueller et al. [51] and Pauwels et al. [52] independently reported the induction of *YUC8* and *YUC9* by OPDA and MeJA. Later, Hentrich et al. [27] clearly demonstrated that wound-induced formation of MeJA in Col-0 Arabidopsis leaves is sufficient to mediate *YUC9* expression. Nevertheless, the molecular mechanism that controls *YUC8/9* expression remained largely uncertain.

In our effort to address whether these two auxin biosynthetic genes are direct targets of the JA signaling pathway, we explored whether MYC2, MYC3, and MYC4 are involved in the transcriptional regulation of *YUC8* and *YUC9*. Our GC-MS/MS and qRT-PCR experiments highlighted that JA-dependent IAA production by *YUC8* and *YUC9* is considerably affected by the presence of the master JA regulator MYC2 (Figure 1 and Figure 2). Our results also disclosed a contribution of MYC3 and MYC4 to MeJA-triggered auxin formation. The latter finding additionally confirms the phylogenetically close relationship of these two bHLH transcription factors with MYC2 (Figure 1). Furthermore, we provide evidence indicating that MYC3 could control the expression of *YUC8*, while MYC4 is probably involved in the transcriptional regulation of *YUC9*. However, since the MeJA treatment induced the accumulation of transcripts of both genes in some *myc* loss-of-function mutants, it may also be possible that alternative transcription factors, like, for instance, MYC5, collaborate in the regulation of *YUC8/9* expression (Figure 2A,B). Extending this hypothesis, it is known that PIF4, which mediates hypocotyl elongation in response to high temperatures, can effectively bind to the G-motif located on the *YUC8* promoter [53]. Furthermore, it has been reported that the jasmonate-inducible ETHYLENE RESPONSE FACTOR 109 (ERF109) physically interacts with the DNA-binding site 5′-GCCGCC-3′ to control *ASA1* and *YUC2* transcript accumulation [54]. However, we were unable to identify the mentioned GCC-box motif in the promoter region of neither *YUC8* nor *YUC9*.

Here, we demonstrated that all three described MYC proteins bind with similar, although not identical affinities to the core 5′-CACGTC-3′ motif, called G-box, and its variants [32,55]. We analyzed the existence of these JA-responsive elements in the promoter sequence of the eleven Arabidopsis *YUCCA* members. Our results clearly identified a specific G-box motif configuration composed by the “tandem” 1-9-1 (5′-CACGTG-CACGTC-CACGTG-3′) followed by the G-box 3 (5′-CATGTG-3′) in the region of the *YUC8* and *YUC9* promoters (Figure 3). We provided multiple lines of evidence, including effector-reporter assays in *N. benthamiana* and *A. thaliana* leaf protoplasts, to demonstrate that all three MYC transcription factors bind to the *YUC8* and *YUC9* promoters in vivo, when the 1-9-1 G-box-tandem or the 3 G-box variants are present (Figure 4 and Figure 5 and Figure A2). Intriguingly, our experiments employing leaf protoplasts further validated that MYC2 acts as a direct regulator of *YUC9* (Figure 5B).

Recently, Santamaría et al. [56] demonstrated that *T. urticae* infestation of Arabidopsis plants activates the MYC2 defense pathway. Taking advantage of this finding, we investigated the biological role of JA-induced IAA biosynthesis by performing a *T. urticae* feeding experiment. As shown in Figure 6A, the pest not only activated the *AOS* promoter, which is known to respond to wounding [57], but also the *YUC9* promoter. Recently, Zhurov et al. [58] reported the significant induction of JA production by *T. urticae* feeding in Arabidopsis Col-0 plants. Moreover, a more recent publication performed in *Nicotiana attenuate* revealed the accumulation of auxin at the site of herbivory by *Manduca sexta* [59]. This localized auxin increase was accompanied by rapid activation of several *YUCCA-like* genes in *N. attenuata*. Thus, our results highlight the importance of the interconnection between JA and IAA through the modulation of *YUC9* expression in plant defense responses. Moreover, the *T. urticae* infestation experiments showed that the auxin overproducer line, YUC9ox, exhibited reduced plant damage, H_2_O_2_ accumulation and cell death in comparison to similarly treated wild-type plants (Figure 6B and Figure A3). It has been demonstrated that the feeding of *T. urticae* on plant leaves proceeds via the insertion of their stylet between the pavement cells or through the open stoma [60]. Thus, it may be speculated that epidermal cell expansion is one reason for the observed enhanced tolerance by limiting spider mite feeding, rather than IAA-mediated immune activation. Congruent with this hypothesis, it has been demonstrated that the transient overexpression of *YUC9* in *N. benthamiana* leaves resulted in significantly expanded pavement cells [27]. Alternatively, it is known that IAA and the biotic stress-related hormone ethylene, can interact at multiple levels [61]. For instance, earlier studies showed that IAA stimulates ethylene biosynthesis through the action of *ACC-SYNTHASE* genes (*ACS*) [62,63,64,65,66]. Likewise, Hentrich et al. [67] observed that YUC8ox and YUC9ox lines are characterized not only by the upregulation of a group of genes related to ethylene production and signaling genes, but also by elevated lignin contents relative to wt, as shown by a qualitative phloroglucinol stain for lignin. Therefore, we suggest that the JA-IAA-ET induced lignification contributes to complicate mite feeding or reduce palatability, probably by an augmentation of the leaf rigidity and a reduction of leaf nutritional values. This hypothesis is particularly supported by the observation that the *T. urticae* mites actively left YUC9ox leaves, which may indicate that the spider mites completely avoid feeding on those leaves.

## 4. Material and Methods

### 4.1. Plant Material 

All presented experiments used the *Arabidopsis thaliana* ecotype Columbia (Col-0) as genetic background (NASC stock N1092). The Arabidopsis *myc* mutants, i.e., *myc2*, *myc3*, *myc4*, *myc2/myc3*, *myc2/myc4*, *myc3/myc4*, and *myc2/myc3/myc4*, the *YUC9* overexpression line YUC9ox, the T-DNA insertion mutant *yuc9ko*, the reporter lines *pAOS::GUS*, *pYUC8::*GUS and *pYUC9::GUS* have been previously described elsewhere [27,32,57,67]. For the sterile growth of plants, seeds were surface sterilization and then stratified at 4 °C for 48 h in darkness. Thereafter, the seeds were sown on solidified ½-strength Murashige and Skoog medium supplemented with 1% sucrose. Plant growth was performed under controlled conditions (22 °C, 16 h light/8 h dark and 100 μmol/m^2^ s^1^ light intensity). For plant defense experiments and protoplast isolation, 10-days old Arabidopsis plants were transferred to a mixture of peat and vermiculite (3:1), and further grown under the same condition described above. The transactivation assay was carried out using 14-days old *N. benthamiana* seedlings grown on peat-based soil under controlled conditions (25 °C and 40–65% relative humidity, 16 h light/ 8 h dark) for 2 to 3 weeks.

### 4.2. qRT-PCR Analysis

To quantify gene expression levels of *YUC8* and *YUC9*, we incubated 10-days old Arabidopsis seedlings with either MeJA (50 µM) or a control mock solution (0.5% methanol, *v*/*v*) over 2 h (*YUC9*) or 4 h (*YUC8*) to account for the different expression strength and response characteristics of the two genes towards MeJA reported by Hentrich et al. [27]. Thereafter, total RNA was isolated from 100 mg of whole seedlings using the phenol:chloroform method, coupled to lithium chloride precipitation, according to Box et al. [68]. The polyA-mRNA was additionally purified using the Oligotex mRNA mini Kit (QIAGEN, Hilden, Germany). Purified mRNA was reverse-transcribed into complementary DNA (cDNA) employing the RNA-dependent DNA polymerase M-MLV (Promega, Madison, WI, USA) following the manufactured instructions. Quantitative RT-PCRs were carried out using a LightCycler^®^ 480 (Roche Diagnostics, Rotkreuz, Switzerland) thermocycler following the manufacturer’s instructions [95 °C for 10 s, 60 °C for 20 s, 72 °C for 30 s] × 45 cycles. For data accuracy, three independent biological replicates were tested in triplicate (technical replicates). The relative gene expression levels were calculated according the 2^−ΔΔCt^ method [69,70]. Primers used for analyzing mRNA levels are listed in Table A1. For data normalization we selected *APT1* and *UBI10* as the reference genes [71].

### 4.3. Auxin Quantification

Extraction of IAA was carried out according to Pérez-Alonso et al. [72]. In essence, approximately 100 mg of 10 days-old seedlings were harvested and directly transferred into 1 mL of methanol containing 50 pmol of the internal standard [^2^H_2_]-IAA (OlChemIm Ltd., Olomouc, Czech Republic). After hormone extraction, the IAA contents were examined by gas GC-MS/MS. For this, dried samples were resuspended in 20 µL derivatization solution (88% acetone:methanol (9:1, *v*/*v*), 11.8% diethyl ether, 1.2% Trimethylsilyl diazomethane, 2 M in diethyl ether). After an incubation of 30 min at RT, 1 µL of the derivatized sample was injected splitless into a BRUKER Daltonics (Bremen, Germany) 451 gas chromatograph equipped with a stationary phase ZB-35 (30 m × 0.25 mm, 0.25 µm film) fused silica capillary column (Phenomenex, Torrance, CA, USA). Helium at a flow rate of 1 mL min^−1^ was used as the mobile phase for the gas chromatographic separation. The injector temperature was set to 250 °C and the column was held at 50 °C for 1.2 min. Thereafter, the temperature was increased by 30 °C min^−1^ to 120 °C, and finally to 325 °C by 10 °C min^−1^ and held there for four additional minutes. The column effluent was introduced into the ion source of a Scion-TQ triple quadrupole mass spectrometer (BRUKER Daltonics, Bremen, Germany). The transfer line and the ion source temperatures were maintained at 250 °C and 200 °C, respectively. Ions were generated by a 70 eV electron beam at an ionization current of 80 µA, and 30 spectra s^−1^ were recorded in the mass range of 50 to 600 *m*/*z*. Under the given conditions the retention time for the endogenous methylated-IAA hormone was 13.6 min. For quantification, we selected the following precursor ions and corresponding diagnostic product ions—MeIAA (*m*/*z* 189/130) and [^2^H_2_]-MeIAA (*m*/*z* 191/132).

### 4.4. In Silico Analysis of YUCCA Promoter Sequences

The 3000 bp promoter regions for all *A. thaliana YUCCA* genes were retrieved from the NCBI database (https://www.ncbi.nlm.nih.gov/gene/, accessed on: 8 September 2021) using the corresponding gene accession numbers: At4g32450 (*YUC1*), At4g13260 (*YUC2*), At1g04610 (*YUC3*), At5g11320 (*YUC4*), At5g43890 (*YUC5*), At5g25620 (*YUC6*), At2g33230 (*YUC7*), At4g28720 (*YUC8*), At1g04180 (*YUC9*), At1g48910 (*YUC10*), At1g21430 (*YUC11*). MYC2 binding motifs in the *YUC* promoter sequences were predicted by running target sequences against known *ci*s-regulatory elements in the AtPan collection (http://plantpan.itps.ncku.edu.tw/, accessed on: 8 September 2021) [73] and PlantCare (http://bioinformatics.psb.ugent.be/webtools/plantcare/html/, accessed on: 8 September 2021) [74] databases. To ensure the incorporation of all the possible G-box variants described by Fernández-Calvo et al. [32] the promoter sequences were also manually inspected.

### 4.5. Transient Expression Analysis in Nicotiana Benthamiana

The *YUC8* and *YUC9* promoter sequences, as well as the coding sequences from MYC2, MYC3, and MYC4 were amplified using PCR specific primers (Table A1) and introduced into the entry vector pSP-Entry1 [75]. Subsequently, *pYUC8/9::GUS* and *35S::MYC2/3/4* constructs were obtained by transferring the target DNA fragments into the destination vectors pMDC-163 [76] or p35S-HA-GW [77,78] by LR clonase reactions (Invitrogen|Thermo Fisher Scientific, Waltham, MA, USA). Subsequently, the *A. tumefaciens*-mediated transient expression experiment was performed according to Ma et al. [79]. In brief, The *Agrobacterium* strain C58C1, carrying the desired construct, and the *Agrobacterium* strain P19, carrying the suppressor of gene silencing from tomato bushy stunt virus (TBSV), were infiltrated into three to four weeks-old *N. benthamiana* plants. Three days post inoculation, the infiltrated leaves were collected and the β-glucuronidase (GUS) activity was determined by histochemical analysis as detailed by Jefferson et al. [80]. 

GUS expression levels were additionally quantified using a fluorometric analysis [81]. For this purpose, two leaf discs were frozen in liquid nitrogen (N_2_), ground and re-suspended in 150 µL of GUS extraction solution [50 mM sodium phosphate buffer Na_2_HPO_4_/NaH_2_PO_4_ pH 7.5, 10 mM EDTA, 0.1% (*v*/*v*) Triton X-100, 0.1% (*w*/*v*) sodium lauroylsarcosinate (Sigma-Aldrich, St. Louis, MO, USA), 0.05% (*v*/*v*) β-MeEtOH]. An aliquot of 10 µL was used for total protein content measurement [82] using bovine-γ-globulin as the protein standard (Bio-Rad Laboratories, Hercules, CA, USA). Whereas an aliquot of 100 µL of the suspension was mixed with 100 µL GUS extraction solution containing 4 mM of 4-methylumeliferyl-β-D-glucuronide (4-MUG) (Duchefa, Haarlem, Netherlands). Samples were then incubated at 37 °C in the dark for 10 min. After the incubation, 100 µL of the 4-MUG solution were separated and the reaction was stopped by the addition of 100 µL of 200 mM Na_2_CO_3_ (T_0_). The remaining 100 µL were further incubated at 37 °C in darkness for 1 h and the reaction was stopped (T_60_). Then, fluorescence was registered at 360 nm excitation and 460 nm emission (56 gain, 10 flashes, 50% mirror) using a TECAN Genios Pro fluorescence spectrometer (MTX Lab Systems, Vienna, VA, USA). The GUS activity was calculated as follows Equations (1) and (2):

(1) $$GUS-Activity\;\left[pmol/min\right]=\frac{\Delta F/10}{t}\;\;$$

(2) $$\;GUS\;Activity=\frac{GUS-Activity}{\;\mathrm{mg}\;\mathrm{of}\;\mathrm{total}\;\mathrm{protein}}$$

where Δ*F* is the difference in fluorescence intensity T_60_-T_0_, 10 are the fluorescence units corresponding to 1 pmol of hydrolyzed 4-MUG and *t* is incubation time. Two independent experiments were carried out and GUS activity was quantified in triplicates.

### 4.6. Arabidopsis Protoplast-Based Transient Expression

To generate the reporter plasmids *pYUC8::GUS* and *pYUC9::GUS*, we amplified the promoter sequences of *YUC8* and *YUC9* containing different MYC2/3/4 binding sites, using PCR specific primers (able A1) and ligated them into the pGEM^®^-T vector (Promega, Madison, WI, USA). Thereafter, DNA fragments were digested by restriction endonucleases and cloned into the pBT-10 plasmid [83]. On the other hand, the effector plasmids *35S:MYC2/3/4* were made as described above. In this case, however, pEarlyGate-210 [84] was used as the destination vector. After construct generation, mesophyll protoplast isolation and PEG-calcium mediated DNA transfection were performed according to Mathur and Koncz [85], Yoo et al. [86], and Alonso et al. [87]. In this work, 9 µg of each reporter construct and 14 µg of the different effectors were utilized. Moreover, to normalize the transfection efficiency, 3 µg of the 35S::neuroaminidase (NAN) plasmid [81] were used. Then, GUS transactivation was quantified by fluorometric analysis as already described. Furthermore, NAN activity was determined according to Kirby and Kavanagh [81]. To do this, from the 150 µL resuspended protoplasts in GUS extraction solution a 10 µL aliquot was mixed with 10 µL NAN extraction solution [50 mM N_2_HPO_4_/NH_2_PO_4_ pH 7.0, 10 mM EDTA, 0.1% (*v*/*v*) Triton X-100, 0.1% (*w*/*v*) sodium lauroylsarcosinate] containing freshly added 0.05% (*v*/*v*) β-MeEtOH and 1 mM 2′-(4-methylumbelliferyl)-α-D-*N*-acetyl-neuroaminic acid (4-MUN) (Duchefa, Haarlem, Netherlands). The protoplasts were then incubated at 37 °C in the dark for 10 min (T_0_). After the incubation, 3.3 µL of the protoplast suspension was transferred to a 200 µL of NAN stop solution [330 mM Na_2_CO_3_]. The remaining protoplast/4-MUN solution was incubated at 37 °C in darkness for 1 h (T_60_). Afterwards, the fluorescence was measured as described before. NAN activity was calculated as previously described [88] Equations (3) and (4):

(3) $$\;\;NAN-Activity\;\left[pmol/min\right]=\frac{\Delta F/10}{t}$$

where Δ*F* is the difference in fluorescence T_60_-T_0_, 10 are the fluorescence units corresponding to 1 pmol of hydrolysed 4-MUN and *t* is the time of incubation. Normalization of the GUS-activity was performed by calculating the ratio of GUS and NAN activities, represented as relative GUS/NAN units, following the Equation (4):

(4) $$\frac{GUS}{NAN}-Activity=\frac{GUS-Activity}{\;NAN-Activity}$$

To ensure data accuracy, GUS and NAN activities were measured in triplicates and each experiment was repeated at least twice.

### 4.7. Plant-Arthropod Interactions

Adult female *T. urticae* spider mites, London strain, isolated from infested bean plants, were carefully placed on the leaf surface from three to four-weeks-old *A. thaliana* plants according to Santamaría et al. [56]. The mites fed for four days in growth chambers (25 °C, 70% relative humidity and with 16 h light/8 h dark regime. Histochemical analyses of GUS activity were performed as described by Jefferson et al. [80]. For leaf damage quantification whole rosette of infested and control plants were scanned using a resolution of 1200 dpi. Plant damage was assessed as the total area of chlorotic spots based on scanned leaves overlaid with a grid of 0.25 mm × 0.25 mm using Adobe Photoshop CS5 software (Adobe Systems, San Jose, CA, USA). For this, all grid units that showed at least 50% damaged areas were marked with a dot of defined size (52 pixels/dot). After marking all damaged areas, the histogram tool was used to quantify the number of pixels on the grid layer. Since each dot is represented by a defined number of pixels, the total number of dots can be calculated by dividing the total number of pixels by the number of pixels per dot. Finally, the total area of damage is calculated according to the following Equation (5):


(5) $$\;\;\;Damage\;area\;\left[mm^{2}\right]=number\;of\;dots\times \;unit\;area\;$$

The damaged area can be calculated in this way because each dot corresponds to one grid unit [89]. We assessed plant damage in five infested independent samples from each genotype. The previously described promoter of the wounding responsive *ALLENE OXIDE SYNTHASE* (*AOS*) gene fused to the GUS reporter gene (*pAOS::GUS*) [57] was used as a positive control in this experiment.

### 4.8. Statistics

The data were analyzed with Student’s *t*-test when two means were compared. Statistical analyses were realized employing the STATGRAPHICS^®^ Centurion XVI (Statpoint Technologies, INC., Warrenton, VA, USA).

## Data Availability

All data supporting the findings of this study are available within the paper and its supplementary data in the appendices.

## Appendix A

Method A1. Trypan Blue staining. Three to four weeks-old, infested *A. thaliana* leaves were harvested after four days of *T. urticae* feeding and stained with 5 mL lactophenol-trypan blue solution (10 mL lactic acid, 10 mL phenol, 10 mL glycerol, 10 mL tryptan blue (Sigma-Aldrich, St. Louis, MO, USA), dissolved in 10 mL of distilled H_2_O) [90]. Before its use, the TB solution was diluted 1:2 with 100% ethanol. The solution including two leaves was then boiled for 1 min and distained for 30 min at room temperature in 2 mL chloral hydrate solution (5 g of chloral hydrate dissolved in 2 mL distilled water). After overnight decolorization, the chloral hydrate solution was removed and then 2 mL 50% glycerol were added. Leaves were then placed on a microscope slide, covered with a cover slip, and analyzed under bright-field lighting using a light stereomicroscope Leica MZ10F (Leica Microsystems, Wetzlar, Germany) at a magnification of ×8 and ×40, respectively. Images were captured using a Leica DFC 400C camera (Leica Microsystems, Wetzlar, Germany). Trypan blue staining was performed for five spider mite infested plants from each genotype and two control plants.

Method A2. DAB staining. We examined H_2_O_2_ accumulation in three to four *A. thaliana* leaves exposed to 4 days of *T. urticae* feeding using the 3,3’-diaminobenzidine (DAB) staining method [91]. For this purpose, two leaves were placed in a 15 mL Falcon tubes, covered with 5 mL DAB solution (0.1% (*w*/*v*) of 3,3-diaminobenzidine-HCl (pH 3.8)), vacuum infiltrated for 5 min and incubated overnight. After incubation, the DAB solution was supplemented with 10 mM ascorbic acid. Then, three subsequent washing steps with 5 mL ethanol/acetic acid/glycerol (*v*/*v*, 3:1:1) of 2 h each were performed to clear leaf tissues. Then, 2 mL of 50% glycerol were added. Microscopy and imaging were carried out using a light stereomicroscope Leica MZ10F (Leica Microsystems, Wetzlar, Germany), at a magnification of ×8 and ×40, and a Leica DFC 400C camera (Leica Microsystems, Wetzlar, Germany). For this experiment five spider mite infested plants and two control plants were used from each genotype.

## Appendix B

**Table A1:** List of primers used for cloning and qRT-PCR analysis (https://quantprime.mpimp-golm.mpg.de/, accessed on: 8 September 2021) [93].

Primer Name	Primer Sequence (5′-3′) and Restriction Sites
Promoter YUC8-(919) For	TATGGATCCAAAAGTGCAGCGTCTACCAAAA
Promoter YUC8-(919) Rev	TATTCTAGATTAGGTACGGAAAATGTGATT
Promoter YUC8-(3) For	TATGGATCCTCCGTACCTAAAAATTGGATT
Promoter YUC8-(3) Rev	TATTCTAGATGCTTGACGACGAAGTAATAAT
Promoter YUC8-(Ø) For	TATGGATCCTCGTCGTCAAGCATTATCACTGTT
Promoter YUC8-(Ø) Rev	TATCCATGGTCTAGATGGAAGTTGTATTGGAAATGGTTT
Promoter YUC9-(919) For	TATAAGCTTAACAAAATTAGGACCCGCTCT
Promoter YUC9-(919) Rev	TATTCTAGAGATTGAATTATATGGTAAACTCAA
Promoter YUC9-(3) For	TATAAGCTTACCACGAAGAAAATAACATCTC
Promoter YUC9-(3) Rev	TATCCATGGTCTAGAGTTAAGAGTTATAACGAGACTG
Promoter YUC9-(Ø) For	TATAAGCTTCAAATTATTCACATTAATAAAATAATC
Promoter YUC9-(Ø) Rev	TATCCATGGTCTAGATTTCTTGAGTGAGTTTTTGAATG
ORF MYC2 For	TATGGTACCATGACTGATTACCGGCTACAACCAACGA
ORF MYC2 Rev	TATGCGGCCGCTTAACCGATTTTTGAAATCAAACTTGCTCTGA
ORF MYC3 For	TATGGATCCATGAACGGCACAACATCATCA
ORF MYC3 Rev	TATGATATCTCAATAGTTTTCTCCGACTTTCGT
ORF MYC4 For	TATGGATCCATGTCTCCGACGAATGTTCAAGTAACCGA
ORF MYC4 Rev	TATGATATCTCATGGACATTCTCCAACTTTCTCCGTT
pENTRY-SP1 For	TATCTGATAGTGACCTGTTCGTTGCA
pENTRY-SP1 Rev	TATGGAGATCCGTGACGCAGTAGC
pBT-10-Seq Rev	TATTTGGGGTTTCTACAGGACGGACCAT
pENTRY-SP1 Rev	TATGGAGATCCGTGACGCAGTAGC
YUC8-qPCR For	CGTCTCAAGCTTCACCTTCC
YUC8-qPCR Rev	AGCCACTGGTCTCATCGAAC
YUC9-qPCR For	TTCTCGCCACCGGTTATCGTAG
YUC9-qPCR Rev	AGCGATGTTAACGGCGTCTACTG
APT1-qPCR For	TCGTGCTGTTCCTTGCAACCG
APT1-qPCR Rev	GCGGAGGAGAAGAGGCGGAGT
UBI10-qPCR For	TTGGAGGATGGCAGAACTCTTGCT
UBI10-qPCR Rev	AGTTTTCCCAGTCAACGTCTTAACGAAA
